# Editorial: The wetware credentials of intermediate filaments involves coordinating, organising and networking in cells and tissues

**DOI:** 10.3389/fcell.2023.1146618

**Published:** 2023-02-13

**Authors:** Rudolf E. Leube, Roy A. Quinlan

**Affiliations:** ^1^ Institute of Molecular and Cellular Anatomy, RWTH Aachen University, Aachen, Germany; ^2^ Department of Biosciences, University of Durham, Upper Mountjoy Science Site, Durham, United Kingdom; ^3^ Biophysical Sciences Institute, University of Durham, Durham, United Kingdom; ^4^ Department of Biological Structure, University of Washington, Seattle, WA, United States

**Keywords:** cytoskeleton, intermediate filament, cell junctions, cell shape, cell polarity and migration, wetware

In this special issue there is a collection of articles that highlight the mechano-biological signalling of and the integration of intermediate filaments within the cytoskeletal machinery. The individual and collective contribution of the individual cytoskeletal elements have been well documented (Ge et al., 2020; Serres et al., 2020; Lois-Bermejo et al., 2022; Nunes Vicente et al., 2022; Ridge et al., 2022; Sivagurunathan et al., 2022; Wu et al., 2022). Indeed the importance of the cytoskeleton as an integrated unit is accepted fully in the literature (Pegoraro et al., 2017; Hohmann and Dehghani, 2019). Intermediate filaments interconnect all subcellular compartments and they are the one cytoskeletal element where cross-β-interactions form intracellular hydrogels (Kato and McKnight, 2018) by virtue of their N- and C-terminal intrinsically disordered domains ((IDDs) (Kornreich et al., 2015))—or plainly put—assist their assembly and their associated phase separation events e.g., (Li et al., 2020). It is no coincidence that previously noted “zones of exclusion” observed by conventional transmission electron microscopy (Blose and Chacko, 1976; Borenfreund et al., 1980) should now be interpreted as evidence of their hydrogel potential e.g., nuclear pores (Fiserova et al., 2014) and cytoplasmic intermediate filament networks (Kornreich et al., 2015). The importance of these IDDs to cell behaviour and to their emergent properties (Ridge et al., 2022) is a hot Research Topic in current debate. This has given rise to exciting hypotheses to explain complex cell behaviours such as motility (see the contributions by Infante and Etienne-Manneville; Kim et al., in this research topic issue) cell polarisation (ibid Despin-Guitard et al.,) epithelial-mesenchymal transitions and inflammatory responses (Ridge et al., 2022).

**FIGURE 1:**
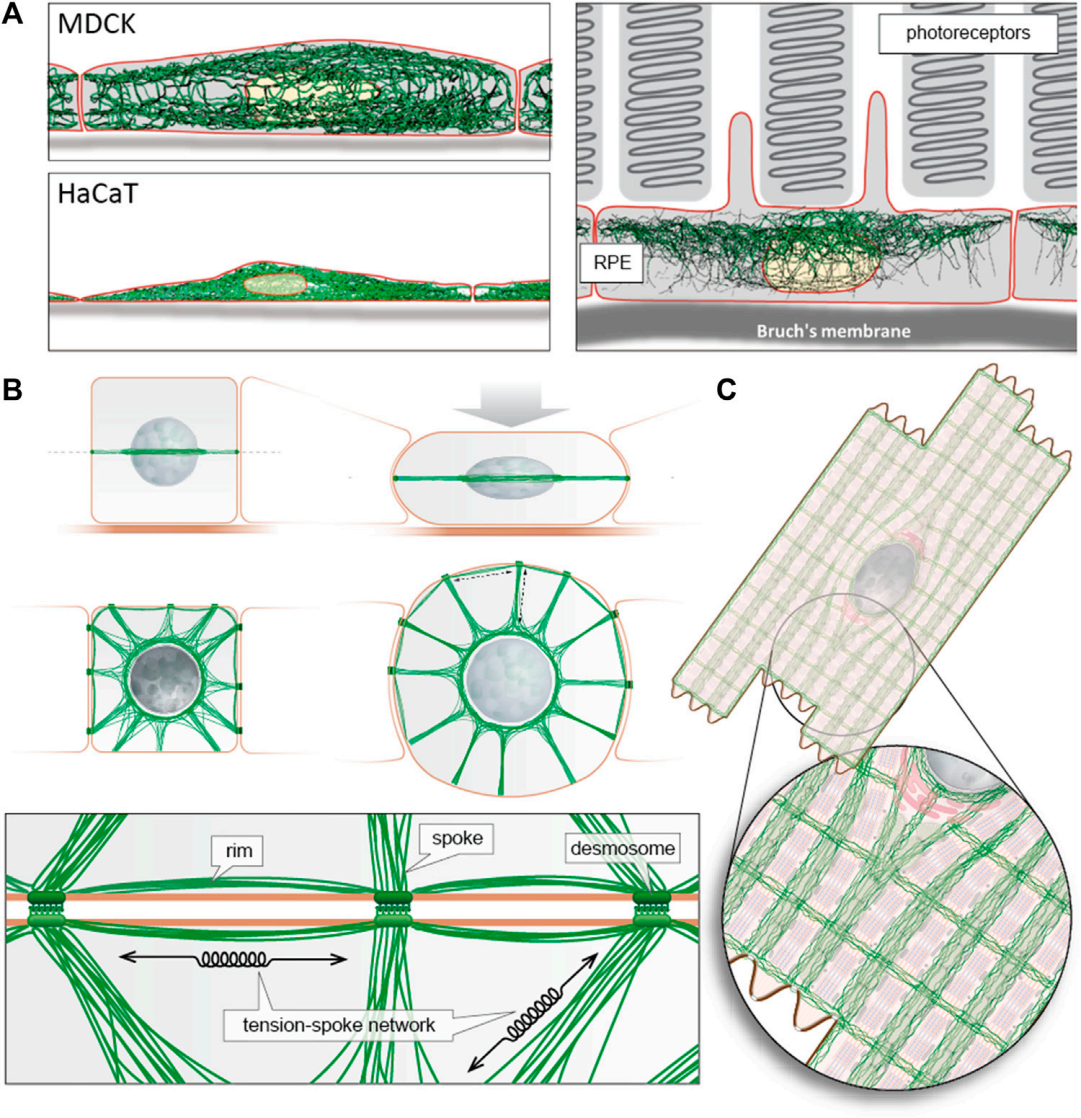
Intermediate filament architecture is cell type specific to support tissue mechanics and function. **(A)** The digital reconstructions of intermediate filament networks that are superimposed on schematic drawings of the corresponding cells are taken from Windoffer et al. (2022). They were derived from 3D recordings of fluorescently labelled keratins in polarized canine MDCK cells, human immortalized epidermal HaCaT keratinocytes and murine retinal pigment epithelial cells *in situ*. Note the different network distributions ranging from apical enrichment (RPE) to apical and basal enrichment (MDCK) and pan cytoplasmic (HaCaT). **(B)** The modified scheme from Quinlan et al. (2017) highlights the circumferential rim and radial spokes arrangement of keratin intermediate filaments connecting the network to the perinuclear cage and adjacent cells through desmosomes. The resulting transcellular tension-spoke system provides mechanical resilience. **(C)** Schematic representation of desmin intermediate filament architecture in cardiomyocytes providing defined subcellular spaces for the ordered contractile apparatus, mitochondria, the nucleus and attachment sites to neighbouring cardiomyocytes and the extracellular matrix (adapted from (Behrendt, 1977; Tokuyasu et al. 1983; Wang and Ramirez-Mitchell, 1983)).

One such hypothesis is the “wetware” concept (Bray, 2009) as a way to conceptualise cellular and tissue decision-making at the level of individual components and processes (Kulkarni et al., 2022). Cell Biology compartmentalises systems and structures, but each function within the context of the cell and the tissue require integration and localised responses of metabolic, structural and cellular pathways. The cytoskeleton collectively provides the architecture (Figure 1) that is needed to sense, communicate and respond to the legion of stimuli received at any one time by each individual cell. It, and its associated biomolecules, can deliver the processing logic for the cell because it provides the required connections (Bray, 2009). In this respect, the intermediate filament cytoskeleton is part and parcel of the stress response (Welch et al., 1985; Quinlan et al., 2002; Landsbury et al., 2010; Toivola et al., 2010) and to the transcriptional (Shimi and Goldman, 2014; Nazer, 2022) and to translational regulation (Magin et al., 2007; Kim and Coulombe, 2010; Mohanasundaram et al., 2022), to chaperone mediated autophagy (Bandyopadhyay et al., 2010), to respiratory efficiency (Diokmetzidou et al., 2016) and to cell division (Matsuyama et al., 2013). This identifies intermediate filaments as key interconnectors for subcellular interaction networks (Kulkarni et al., 2022). Indeed, the intermediate filament provides a surface to facilitate biomolecular folding, biomolecular complex assembly and complex organisation. Intermediate filaments as a collective provide a scale-free network across diverse length scales especially as a result of the inter-cellular organisation they afford within a tissue *via* their connection to cell-cell junctions such as the desmosome (see Green et al., in this research topic issue). Their integrative role in mechano-signalling (Infante and Etienne-Manneville; this research topic issue) is well founded and super-resolution microscopy demonstrates that stretching filaments will reveal new, and quite possibly novel, functional nanodomains (Massou et al., 2020; Nunes Vicente et al., 2022) as also shown for lipo-oxidative stress (Lois-Bermejo et al., 2022). Stress reveals the importance of the C-terminal IDDs to the biophysical properties of intermediate filaments (Aufderhorst-Roberts and Koenderink, 2019) as well as to their assembly and ultimately therefore also to cell morphology (Zhou et al., 2021).

The Research Topic highlights aspects of epithelial keratin network organization. Using a Krt8:YFP reporter mouse Desprin-Guitard and colleagues (see Despin-Guitard et al., in this research topic issue) study the keratin intermediate filament network in the developing mouse embryo revealing a kaleidoscope of temporally and spatially determined expression profiles in embryonic and extraembryonic tissues which are interpreted as plastic adaptations of cell mechanics to growth and morphological changes. The review by Green and colleagues in this research topic issue focuses on the epidermal desmosome-keratin system as an integrator of mechanically-determined signalling. In concert with other junctions, desmosomes dictate epidermal polarization and differentiation forming a barrier by stratum-specific junctional and cytoskeletal arrangements. The authors suggest that these arrangements counteract inflammation. The paper by Yoon and colleagues in this research topic issue presents technical advancements for multidimensional and multimodal monitoring of keratin filament architecture and function. High resolution microscopy of fluorescent keratins is enabled on defined matrices and combined with traction force microscopy. In this way, the interrelationship between extracellular matrix cues with global 3D cytoskeletal network properties at the keratin filament/keratin bundle level and local forces is quantified by refined image analysis. It is further illustrated that these tools can be used for monitoring the consequences of local keratin network perturbations and ECM composition on cell mechanics in the context of transcellular network arrangement.

Infante and Etienne-Manneville in this research topic issue summarize current knowledge about the spatial arrangement and integration of cytoplasmic and nuclear intermediate filaments and their interaction with other cytoplasmic filament systems during cell migration. They emphasize the different properties of the different intermediate filament types as a basis of cell type- and function-related cellular mechanics. They further highlight the cooperativity between intermediate filaments with the other cytoskeletal systems determining motile properties of single cells and cell collectives. Direct experimental assessment of vimentin’s function during metastatic invasion is finally provided by Kim and colleagues in this research topic issue. Using a novel vimentin-stabilizing drug they report on altered vimentin network morphology with consequences on adhesion and contractility resulting in cell shape changes, increased tractions forces and perturbed migration.

Figure 1 presents examples of intermediate filament network organization to illustrate their function both as organizers of the subcellular space (Schwarz and Leube, 2016) and as transcellular integrators to facilitate and support coordinated mechanical and biochemical functions in the context of tissues rather than individual cells (Hatzfeld et al., 2017). It is this framework upon which the contributions in this Research Topic have been made.
